# Using Single-Molecule Chemo-Mechanical Unfolding to Simultaneously Probe Multiple Structural Parameters in Protein Folding

**DOI:** 10.3390/mps2020032

**Published:** 2019-04-20

**Authors:** Emily J. Guinn, Susan Marqusee

**Affiliations:** 1 Department of Chemistry and Biochemistry, DePauw University, Greencastle, IN 46135, USA, emilyguinn@depauw.edu; 2 Institute for Quantitative Biosciences (QB3), University of California, Berkeley, CA 94720, USA; 3 Department of Molecular and Cell Biology, University of California, Berkeley, CA 94720, USA; 4 Department of Chemistry, University of California, Berkeley, CA 94720, USA; 5 Chan Zuckerburg Biohub, San Francisco, CA 94158, USA

**Keywords:** force spectroscopy, optical tweezers, chemo-mechanical unfolding, protein folding, denaturant, urea

## Abstract

While single-molecule force spectroscopy has greatly advanced the study of protein folding, there are limitations to what can be learned from studying the effect of force alone. We developed a novel technique, chemo-mechanical unfolding, that combines multiple perturbants—force and chemical denaturant—to more fully characterize the folding process by simultaneously probing multiple structural parameters—the change in end-to-end distance, and solvent accessible surface area. Here, we describe the theoretical background, experimental design, and data analysis for chemo-mechanical unfolding experiments probing protein folding thermodynamics and kinetics. This technique has been applied to characterize parallel protein folding pathways, the protein denatured state, protein folding on the ribosome, and protein folding intermediates.

## 1. Introduction

Advances in single-molecule force spectroscopy over the past decade have created a unique and powerful tool to study protein folding [1,2,3,4]. Traditional protein folding studies use ensemble approaches where the folding equilibrium is perturbed using chemical denaturant, temperature, or pH [5,6,7]. The resulting data are analyzed to characterize the protein energy landscape, the map of energetics and dynamics for the ensemble of possible protein conformations [8,9,10]. Single-molecule force experiments have revealed the role of mechanical stress on this energy landscape and opened up new avenues to probe unique aspects of the landscape. For instance, the ability to follow the conformational trajectory of single protein molecules provides insight into the inherent heterogeneity of the folding process [11,12,13,14]. Additionally, because the tethers used to apply force can be attached in different places on the protein, the region of the protein that is perturbed by the force vector can be varied [13,15,16]. Finally, single-molecule force experiments can be used to characterize how proteins behave in the context of larger molecular machines such as during protein synthesis and degradation [2,17,18,19].

Different perturbants alter protein conformations by selectively stabilizing/destabilizing different structural features of a protein. The degree to which a conformational change depends on the perturbant reveals specific structural information about the landscape. In single molecule force experiments, the force dependence of the reaction can be used to determine a structural parameter called the ***x***-value. This ***x***-value describes the change in end-to-end distance of the protein along the pulling axis during a conformational change like folding or unfolding and can, therefore, be used to characterize and compare folding trajectories in terms of changes in extension [1,19]. Similarly, the denaturant dependence of a reaction is used to determine a parameter called the *m*-value, which characterizes the solvent-accessible surface area buried or exposed during a conformational change [20,21].

Although extremely useful, there are limitations to what can be gained from measuring a single parameter such as the *x*-value. For instance, because most techniques used to monitor protein conformational changes do not measure extension changes, it is difficult to compare single-molecule force data to data collected using more traditional techniques. Moreover, because the extension changes determined from force experiments are related to the specific pulling axis used, data and resulting *x*-value obtained along one pulling axis cannot be compared directly to those from a different pulling axis.

Here, we describe a recently developed technique called chemo-mechanical unfolding that combines force and denaturant to extend and enhance the information that can be obtained using the optical tweezers [12,13,22]. Combining multiple perturbants, such a chemical denaturant and force, permits determination of multiple structural parameters for the same process. This allows us to more fully characterize the folding process, identify folding trajectories over multiple barriers, and use the added parameter to compare folding pathways across different experiments and pulling axes.

## 2. Experimental Design

### 2.1. Structural Parameters Used in Chemo-Mechanical Unfolding

**Figure 1:**
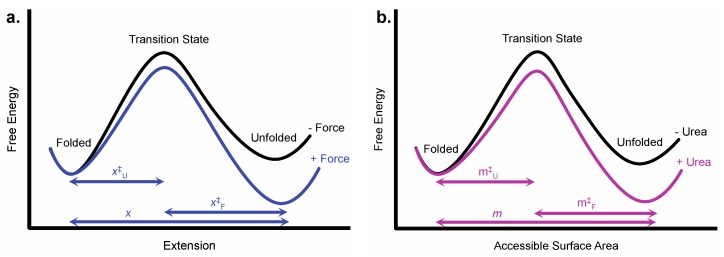
Effect of force and urea on a one-dimensional protein energy landscape. (**a**) Extension, which is related to the *x*-value, is the reaction coordinate determined from the effect of force on protein unfolding. Force preferentially lowers the free energy of more extended conformations like the transition state and the unfolded state. (**b**) Accessible surface area, which is related to the *m*-value, is the reaction coordinate determined from the effect of urea on protein unfolding. Urea preferentially lowers the free energy of conformations that expose more accessible surface area.

Perturbants like chemical denaturant and force denature proteins by selectively destabilizing the protein’s native state compared to other states such as the denatured state (Figure 1). The magnitude of this perturbation is related to the magnitude of the change in some conformational property of the two states. For example, the chemical denaturant urea interacts favorably with the amide and hydrocarbon surface that is generally exposed in the unfolded state and buried when the protein folds [21]. Urea, therefore promotes unfolding by promoting the exposure of this surface to solvent in the unfolded state. Urea has a stronger denaturing effect on proteins that bury more amide and hydrocarbon surface upon folding.

Perturbants can also alter the position of states along the reaction coordinate if they affect the structure of these states. For instance, force promotes a more extended denatured state, thus shifting the position of the unfolded state on the extension reaction coordinate [19]. On the other hand, recent work suggests that the accessible surface area of the denatured state does not vary with force or urea concentration so the unfolded state will not shift significantly along the accessible surface area reaction coordinate [12]. We do not take into account such potential changes in position along the reaction coordinate in our chemo-mechanical unfolding analysis.

We quantify the effect of urea on protein folding thermodynamics and kinetics using a parameter called the *m*-value. For instance, urea’s thermodynamic *m*-value quantifies the effect of urea on ΔG_U_, the change in free energy for unfolding a protein, via Equation (1):

(1) $$\Delta G_{U}=\Delta G_{U}^{0M\;\mathrm{urea}}-m\left[\mathrm{urea}\right]$$

where Δ*G_U_*^0Murea^ is the change in free energy in the absence of urea and [urea] is the molar concentration of urea [20,23]. These thermodynamic *m*-values are directly correlated with the change in accessible surface area (ΔASA), the amount of accessible surface area of the protein exposed in the transition from the folded state to the unfolded state [20,21]. Therefore, quantifying the effect of urea on ΔG_U_, yields information about the conformational change of the protein in terms of ASA.

Similarly, kinetic *m*^‡^-values for folding (*m_F_*^‡^) and unfolding (*m_U_*^‡^) quantify the effect of urea on the folding (*k_F_*) or unfolding (*k_U_*) rate constant respectively via Equation (2):

(2) $$lnk_{UorF}=lnk_{UorF}^{0M\;\mathrm{urea}}-m_{UorF}^{‡}\left[\mathrm{urea}\right]$$

where *k_UorF_*^0Murea^ is the unfolding or folding rate constant in the absence of urea. Just as the thermodynamic *m*-value is related to ΔASA for unfolding, kinetic *m*^‡^-values are related to ΔASA for folding or unfolding to the high-free energy transition state [24,25]. The *m_F_*^‡^-value is proportional to ΔASA for the transition from the unfolded state to the transition state and the *m*_U_^‡^-value is proportional to ΔASA for the transition from the folded state to the transition state. Therefore, by measuring the effect of urea on protein folding thermodynamics and kinetics, we can get useful information about the relative ASA buried in the folded state, transition state, and unfolded state, providing insight into the conformation of the folded protein and the transition state.

The effect of force can also be related to the structural properties of protein conformations. Force unfolds a protein by biasing it to more extended states. In optical tweezers experiments, force is applied along a specific axis that is defined by the point of attachment of the tethers used to pull the protein (see the section below for details on mechanical unfolding with the optical tweezers). The effect of force is related to the change in distance or extension between those points in the folded and unfolded state [19]. The energetics and kinetics of a process with a larger change in extension will be more significantly affected by force; if there is no change in extension between the points then the force will have no effect on the process.

Analogous to urea *m*-values, the *x*-value describes the effect of force on protein folding thermodynamics and kinetics. The Bell model gives Equations (3) and (4), which, similar to Equations (1) and (2), relate the thermodynamic *x*-value and the kinetic *x*^‡^-values (*x*_U_^‡^ and *x*_F_^‡^) to ΔG, *k*_U,_ or *k*_F_ and the force applied (F):

(3) $$\Delta G_{U}=\Delta G_{U}^{0\mathrm{pN}\;\mathrm{Force}}-F\frac{x}{k_{B}T}.\;$$

(4) $$lnk_{UorF}=lnk_{UorF}^{0\mathrm{pN}\;\mathrm{Force}}-F\;x_{UorF}^{‡}/\;k_{B}T$$

where Δ*G_U_*^0pNForce^ is the change in free energy in the absence of force and *k*_U*orF*_^0pNForce^ is the unfolding or folding rate constant in the absence of force, T is the absolute temperature and *k_B_* is Boltzmann’s constant [1,19,26]. According to the Bell model, *x* is the distance between the folded and unfolded state, *x_U_*^‡^ is the distance between the folded state and the transition state and *x_F_*^‡^ is the distance between the unfolded state and transition state (Figure 1). The Bell model does a good job describing the effect of force on stability and rates of many proteins, but sometimes proteins deviate from Bell model behavior and other models are required [27]. The parameters measured in chemo-mechanical unfolding and the structural information that they convey are listed in Table 1.

Force and chemical denaturant each provide important information about different aspects of conformational transition in proteins—extension and surface area. Separately, these parameters are useful, but when combined in what we term chemo-mechanical unfolding experiments, they yield even more information. Chemo-mechanical unfolding experiments simultaneously determine *m*-values and *x*-values (equilibrium experiments) or *m*^‡^-values and *x*^‡^-values (kinetic experiments), providing details about protein folding that could not be obtained from force or chemical denaturation alone.

### 2.2. Chemo-Mechanical Unfolding using Optical Tweezers

**Figure 2:**
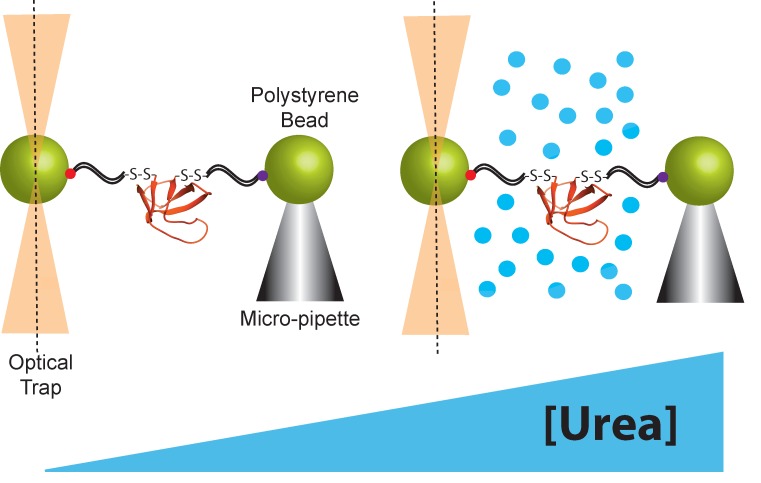
Schematic representation of the optical tweezers set-up used in chemo-mechanical unfolding experiments.

Both atomic force microscopy (AFM) and optical tweezers can be used to manipulate protein molecules mechanically. Here, we limit our discussion to the optical tweezers because they apply force in the low regime (below 60 pN) where proteins can be observed to fluctuate between the folded and unfolded state, allowing us to measure thermodynamics as well as kinetics [1]. Briefly, for protein-folding studies using the optical tweezers, a protein molecule is tethered between two micron-sized polystyrene beads using double-stranded DNA handles (Figure 2). These DNA handles serve as spacers between the bead and a single protein molecule. The DNA is functionalized at each end—one end is attached to the polystyrene beads and the other end is attached to a specific point in the protein, usually through an engineered disulfide bond, although other types of functionalization can also be used. One bead is held in an optical trap that is used to manipulate the bead and apply force to the protein molecule. The other bead is held in either a pipette tip or a second optical trap. Folding and unfolding are monitored by applying force and observing the extension of the protein molecule as a function of time. There are several good reviews for both the sample preparation and the optical trap set up [1,28,29,30,31,32]. The force-dependent kinetics of conformational changes can be determined by: (1) holding the beads at a constant force or trap position, and monitoring the dwell times as the protein fluctuates from one state to the other, or (2) rapidly changing the force (force jump) to change the conformational bias and monitoring the time before a conformational switch [1]. In both of the above cases, the dwell time in each state is used to determine ΔG, *k_U_*, and *k_F_*. Determining these variables as a function of force yields the thermodynamic and kinetic *x*-values. Simultaneously, urea *m*-values are determined from the urea-dependence of ΔG, *k_U_*, and *k_F_*, which is obtained by repeating these experiments in the presence of different concentrations of urea [12,13]. Alternatively, force-dependent kinetics can be determined from the more complicated force-ramp experiment, a far-from-equilibrium experiment where the force is continuously changed. There are several good reviews on how to measure these kinetics and energetics using the optical trap [4,33,34]. More details about how thermodynamic and kinetic chemo-mechanical data are collected and thermodynamic and kinetic parameters are extracted are given below.

### 2.3. Collecting and Fitting Chemo-Mechanical Unfolding Data for Folding Thermodynamics

To monitor folding and unfolding in equilibrium in the optical tweezers, folding and unfolding must be measured at the same force. Generally, folding is more easily observed at lower forces where the folding rate is experimentally observable (at high forces folding will be too slow to observe). Conversely, unfolding is more easily observed at higher forces where unfolding occurs on an experimentally observable timescale. Thermodynamic studies, therefore require a protein where the folding force regime and unfolding force regime overlap [12].

**Figure 3:**
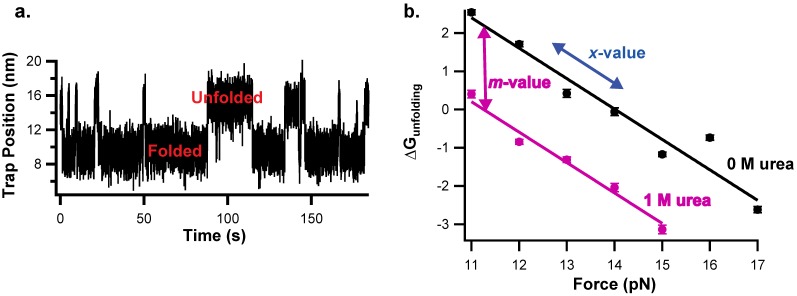
Chemo-mechanical unfolding to explore protein folding thermodynamics of Acyl CoA Binding protein (ACBP, as studied in reference [12], which is described in the applications section below). (**a**) Example of experimental hopping data where ACBP is held at a constant force and observed to hop between folded and unfolded states. (**b**) Chemo-mechanical unfolding data showing ΔG_unfolding_ as a function of force in 0 and 1 M urea. The *x*-value is determined from the slope of ΔG_unfolding_ vs. force and the *m*-value is determined from the slope of ΔG_unfolding_ vs. [urea], or the difference between the 0 M urea and 1M urea data shown here.

The easiest way to monitor folding and unfolding at the same force is to hold the sample at a constant force using force-feedback mode, where a feedback adjusts the trap position to maintain a constant force, and extension is monitored as a function of time. The protein will hop between the folded and unfolded state, observed as an increase in extension when the protein unfolds and a decrease in extension when the protein folds (Figure 3a). This is only suitable for proteins with conformational changes fast enough to sample both states within a reasonable time frame yet slower than the time constant of the force feedback. The resulting extension versus time plots are analyzed to identify individual transitions. For relatively clean data, this can be done by simply drawing a line between the folded and unfolded state extension and marking each time the data cross this line as a transition. However, accurate transitions cannot be identified using this simple model for data with significant noise; a hidden Markov model can be used to detect transitions for noisy data [12,35,36].

For thermodynamic studies, the equilibrium constant (K_eq_) for unfolding can be determined from the ratio of the overall dwell time in each state (K_eq_ = total time unfolded/total time folded). Alternatively, folding and unfolding rate constants can be determined separately using the dwell times in the unfolded and folded state respectively; the ratio of these rate constants is the equilibrium constant (K_eq_ = *k_u_/k_f_*). Finally, the free energy for unfolding is determined from the unfolding equilibrium constant (ΔG = -RTlnK_eq_, where R is the ideal gas constant). These experiments are repeated at a variety of forces to determine ΔG or lnK_eq_ as a function of force. To determine the urea-dependence of ΔG and lnK_eq_, these experiments are then repeated in buffer containing urea (Figure 3b).

Next, the data are fit to determine both the *m*-value and *x*-value. As described by Equation (3), the *x*-value is the slope of ΔG_U_ vs. force. Because these experiments are so labor intensive, often only two buffer conditions are used, 0M urea and 1M urea, resulting in data for ΔG vs. force in both urea concentrations. If urea does not affect the change in extension for unfolding, the slope of these ΔG_U_ vs. force lines should be the same and the 0M and 1M urea *x*-values can be linked in the fit. The urea *m*-value is determined from the urea-dependence of ΔG_U_ described in Equation (1). We can simultaneously fit all the data by combining Equations (1) and (3) in a global analysis:

(5) $$\Delta G_{U}=\Delta G_{U}^{0\mathrm{pN}\;\mathrm{Force},\;0M\;\mathrm{urea}}-m\left[\mathrm{urea}\right]-F\frac{x}{k_{B}T}$$

The resulting fit gives the *m*-value and *x*-value, and therefore the extension change and ASA change, for unfolding. The *m*-value is particularly useful because it can be compared to *m*-values collected in standard ensemble experiments allowing the much-needed comparison between the optical trap and bulk solution. Both parameters give information about the conformational change between the folded and unfolded state and can be used to learn about the structures of these states [12].

### 2.4. Collecting and Fitting Chemo-Mechanical Unfolding Data for Folding Kinetics

**Figure 4:**
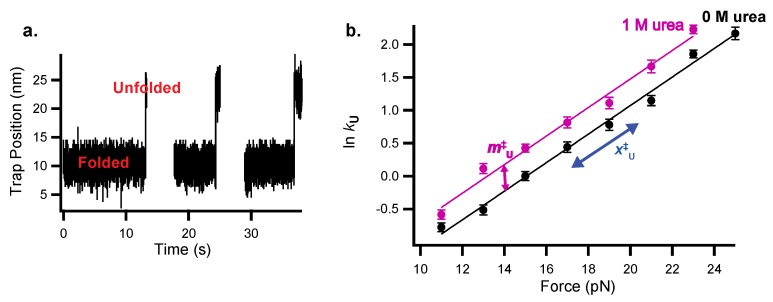
Chemo-mechanical unfolding to explore protein folding kinetics of the src SH3 domain (as studied in reference [13], which is described in the applications section below). (**a**) Example of force-jump data to monitor src SH3 unfolding kinetics where the protein is jumped to a constant force and observed to unfold by monitoring trap position. (**b**) Chemo-mechanical unfolding data showing ln k_U_ as a function of the force in 0 and 1 M urea. The *x*^‡^*_U_*-value is determined from the slope of ln *k_U_* vs. force and the *m*^‡^*_U_*-value is determined from the slope of ln *k_U_* vs. [urea], or the difference between the 0 M urea and 1 M urea data shown here.

Force-jump experiments are used to measure folding kinetics in the optical tweezers for a protein where folding or unfolding is too slow to observe the protein to hop as described above. For unfolding, the protein is initially held at a low force where it is likely to be folded and the force is jumped to a higher force where the protein is likely to unfold (Figure 4a). After the jump, extension vs. time is monitored, and unfolding is observed as an increase in extension.

The dwell time in the folded state is used to determine the unfolding rate constant (***k_u_*** = 1/dwell time). To measure folding, the inverse experiment is performed—the protein is initially held at a high force where it will be unfolded and jumped to a lower force. In practice, folding rates are more difficult to determine because folding occurs at very low forces where the resolution of the optical tweezers is poor.

These folding and unfolding experiments are performed at a range of forces and then repeated as a function of urea (or more simply in 1M urea) to determine the urea dependence of the rate constants as a function of force (Figure 4b). As with the thermodynamic data above, the data can be fit using a global analysis that combines Equations (2) and (4) to simultaneously determine *x*^‡^-values and *m*^‡^-values. Again, if the slopes of lnk vs. force for the 0M and 1M urea data are the same, the 0 M and 1 M urea *x*^‡^-values can be linked in the fit:

(6) $$lnk_{UorF}\;=\;lnk_{UorF}^{0\mathrm{pN}\;\mathrm{Force},\;0M\;\mathrm{urea}}\;-\;m_{UorF}^{‡}\;\left[\mathrm{urea}\right]\;-F\frac{x_{UorF}^{‡}}{k_{B}T}$$

The resulting *m*^‡^-values and *x*^‡^-values give structural information about the protein folding transition state, and so can be used to characterize the folding pathway.

The analysis above applies to a protein with a single rate-limiting barrier (i.e., transition state) under all force and urea conditions studied. However, for many proteins, multiple transition states are possible either due to the presence of an intermediate (multiple barriers on the same pathway) or parallel folding pathways. The effect of force and urea on the height of each barrier depends on the structure of the transition state. It is possible that by selectively destabilizing a certain barrier, force or urea could change which barrier is rate limiting, in which case the slope of the lnk vs. force plot will change reflecting the *x*^‡^-value, or extension, of the new transition state [13].

## 3. Applications of Chemo-Mechanical Unfolding

### 3.1. Chemo-Mechanical Analysis of Unfolding Thermodynamics in ACBP to Probe the Denatured State

The protein denatured state is highly debated—how much structure does the denatured state contain and does it change with environmental conditions? Chemo-mechanical unfolding can be used to answer both questions because this technique not only monitors the structural change involved in unfolding via two parameters (ASA and extension), but also perturbs environmental conditions (force and denaturant concentration). The thermodynamic *m*- and *x*-values are related to the difference in ASA and extension between the folded and unfolded state (*m*-value ∝ ASA_unfolded_—ASA_folded_ and *x*-value ∝ extension_unfolded_—extension_folded_). The folded state does not change significantly with the environment, so any difference in *m*-or *x*-value seen under different force or denaturant conditions represents a change in the ASA or extension of the unfolded state.

Chemo-mechanical unfolding analysis of protein folding thermodynamics has been used to probe folding of the Acyl CoA Binding protein (ACBP) [12]. ACBP folding and unfolding rates are experimentally observable when approximately 15 pN of force are applied [37]; therefore, hopping experiments were used to determine the folding equilibrium constant as a function of the force in 0 M urea and 1 M urea. The resulting data were fit to Equation (5) to determine the *m*-value and *x*-value. The *m*-value was also determined in the absence of force using a standard denaturant melt. Comparison of the *m*-values collected in the presence and absence of force reveals no significant effect of force or urea concentration on the *m*-value. Therefore, under all urea and force conditions studied, the denatured state ASA and extension of the denatured state do not change, indicating that the force-and urea-induced denatured states have a similar structure.

### 3.2. Chemo-Mechanical Unfolding Reveals Parallel Unfolding Pathways in the Src SH3 Domain

Another enduring debate is whether proteins fold through one pathway or many parallel pathways. Chemo-mechanical unfolding can also help answer this question. Kinetic *m*^‡^- and *x*^‡^-values characterize protein-folding pathways by giving structural information about the transition state. Additionally, force and urea may perturb folding enough to switch the dominant pathway. Because force is applied along a specific axis, force can be used to destabilize different regions of the protein and potentially favor alternative folding pathways.

In recent work exploring folding pathways, force was applied to the src SH3 domain along two different pulling geometries (termed the shearing and unzipping geometries) and unfolding kinetics were quantified via force-jump experiments [13,15]. When force was applied in the unzipping direction, ln*k_u_* vs. force plots were linear as described by Equation (4). However, when force was applied in the shearing direction, the plots showed upward curvature. These data can be fit as the sum of two lines which have two different slopes, or *x*^‡^-values, suggesting that src SH3 folds through two different pathways. Analysis of force-dependent folding using an analytical framework verifies that this upward curvature is a signature of parallel folding pathways [38,39]. The chemo-mechanical unfolding analysis was used to characterize these pathways further and compare them to the unzipping pathway and the pathway seen in standard ensemble experiments. The *m*^‡^-values measured in chemo-mechanical unfolding experiments suggest that the unzipping pathway is the same as the pathway in the absence of force (from standard-ensemble experiments), but the pathways seen in shearing experiments represent two new pathways. Therefore, chemo-mechanical unfolding experiments revealed that src SH3 unfolds through at least three unique pathways and that the flux between pathways shifts with relatively small changes in urea and force.

### 3.3. Chemo-Mechanical Unfolding Probes the Effect of the Ribosome on Folding Pathways

Chemo-mechanical unfolding can be used for more complicated systems than isolated protein domains. The optical tweezers have been used to study complex molecular machinery, such as the proteasome, RNA polymerase, and the ribosome [2,17,18,40]. By adding urea to these systems, chemo-mechanical unfolding can be used to gain more information about the conformational changes involved in the processes that these molecular machines catalyze. Using recently developed methodologies [17,41,42], the optical tweezers can be used to apply force to proteins tethered to the ribosome (ribosome nascent chains)—the addition of denaturant to these experiments can provide even more insight into protein folding on the ribosome.

As shown in the previous example, the src SH3 domain unfolds through parallel pathways whose flux depend on environmental conditions. This suggests that the cellular environment could affect the protein folding pathway. In the cell, many proteins fold as they are translated by the ribosome, so the ribosome may alter the folding pathway. To probe if the ribosome alters the flux through the different folding pathways for src SH3, chemo-mechanical unfolding analysis of src SH3 unfolding kinetics was used with src SH3 ribosome nascent chains and free protein [22]. The resulting kinetic *m*^‡^- and *x*^‡^-values were the same for src SH3 on and off the ribosome, indicating that the ribosome does not alter the folding pathway. Therefore, single protein domains may fold through the same pathway as they are being translated that they fold through when free in solution. This work also demonstrates the utility of chemo-mechanical unfolding to study complicated molecular machinery.

### 3.4. Chemo-Mechanical Analysis to Characterize Folding Intermediate in T4-Lysozyme

The chemo- component of the chemo-mechanical analysis is not limited to urea or even to denaturants; chemo-mechanical unfolding experiments can use any solute that perturbs protein folding, including stabilizing osmolytes [43]. Stabilizing osmolytes provide a counter-effect to the denaturing effect of force, allowing higher forces to be accessed in unfolding experiments.

Chemo-mechanical experiments using the osmolytes sorbitol and trimethylamine N-Oxide (TMAO) have been used to study the folding and unfolding pathway of T4 lysozyme, which folds and unfolds through an intermediate [32]. As with urea, osmolyte-effects on folding and unfolding are related to ASA changes during the folding process, so chemo-mechanical analysis can be used to help characterize the structure of these intermediates. Osmolytes did not have a significant effect on unfolding kinetics of T4-lysozyme, but they did significantly increase folding rates. Moreover, the osmolyte m^‡^- and x^‡^-values were used to identify a structural model for the folding intermediate. Different solutes have different strengths of interaction with different types of ASA, so chemo-mechanical unfolding experiments performed with multiple solutes provide increased structural resolution about the types of ASA involved in a process.

## 4. Conclusions

The above examples demonstrate the many different ways that chemo-mechanical unfolding can be used to characterize protein folding. By combining multiple perturbants, chemo-mechanical analysis allows access to new regions of protein energy landscapes, provides additional structural information about the folding pathway, and provides a way to compare force and ensemble data.

## Figures and Tables

**Table 1:** Parameters measured in chemo-mechanical unfolding experiments and the structural information that they provide.

Parameter	Experimental Origin	Structural Information
Urea *m*-value	Urea effect on ΔG_unfolding_	Change in accessible surface area between folded and unfolded state
Urea *m_U_*^‡^-value	Urea effect on unfolding rate constant	Change in accessible surface area between folded state and transition state
Urea *m_F_*^‡^-value	Urea effect on folding rate constant	Change in accessible surface area between unfolded state and transition state
Force *x*-value	Force effect on ΔG_unfolding_	Change in extension between folded and unfolded state
Force *x_U_*^‡^-value	Force effect on unfolding rate constant	Change in extension between folded state and transition state
Force *x_F_*^‡^-value	Force effect on folding rate constant	Change in extension between unfolded state and transition state
